# Transcriptomic and Metabolomic Analysis of Quality Changes during Sweet Cherry Fruit Development and Mining of Related Genes

**DOI:** 10.3390/ijms23137402

**Published:** 2022-07-03

**Authors:** Chaoqun Chen, Hongxu Chen, Wenlong Yang, Jie Li, Wenjing Tang, Ronggao Gong

**Affiliations:** College of Horticulture, Sichuan Agricultural University, Chengdu 611130, China; 2020205005@stu.sicau.edu.cn (C.C.); s20166113@stu.sicau.edu.cn (H.C.); yangwenlong@stu.sicau.edu.cn (W.Y.); lijie@stu.sicau.edu.cn (J.L.); 2020305058@stu.sicau.edu.cn (W.T.)

**Keywords:** sweet cherry, fruit development, transcriptome, metabolome, quality formation

## Abstract

Sweet cherries are economically important fruit trees, and their quality changes during development need to be determined. The mechanism of fruit quality changes in sweet cherries were determined by analyzing sweet cherry fruits at 12 developmental stages. The results showed that the soluble sugar, anthocyanin content, and hormones of sweet cherries all changed drastically during the color transition. Therefore, the fruits at the beginning of color conversion, at the end of color conversion, and at the ripening state were selected for the comprehensive analysis of their metabolome and transcriptome. Different sugars, such as D-glucose, sucrose, and trehalose, were identified in the metabolome. Dihydroquercetin, delphinidin-3-glucoside, cyanidin-3-rutincoside, and other flavonoid species were also identified. D-glucose and cyanidin-3-rutinoside were among the most important components of sweet cherry soluble sugars and anthocyanins, respectively. The transcriptional analysis identified key structural genes and nine transcription factors involved in the ABA, sugar, organic acid, and anthocyanin synthesis pathways, with the following specific regulatory patterns. *NAC71*, *WRKY57*, and *WRKY3* regulate fruit sugar accumulation mainly by acting on *INV*, *SPS*, and *SUS*. *MYC2* is involved in the synthesis of anthocyanin precursors by activating *PAL* and *C4H*, whereas *TCP7* mainly regulates *CHI* and *F3H*. *WRKY3*, *NAC71,* and *WRKY57* have important positive regulatory significance on anthocyanin accumulation, mainly by activating the expression of *DFR*, *ANS*, and *3GT*.

## 1. Introduction

Sweet cherry (*Prunus avium* L.) belongs to the Rosaceae tree [1]. As an important economic fruit tree, it is native to Europe and widely cultivated in China. Sweet cherries contain nutrients such as anthocyanins, vitamins, proteins, and minerals, as well as active substances such as phenols and flavonoids, which help fight cell damage, reduce inflammation, and promote overall health. The growth curve of sweet cherry is of the double ‘S’ type. According to its developmental characteristics, the development process is mainly divided into three stages. The first stage is mainly based on cell division and elongation, when the fruit mainly appears green; the second stage is the endocarp hardening stage, which results in the formation of the nucleus and the color formation of the fruit; the third stage is the exponential growth period caused by cell enlargement, in which physiological and biochemical changes drastically occur in sugar, organic acid, and color [2]. In the process of fruit development, fruit quality is an important reference for determining the nutritional value and commercial value of fruit, and it is mainly divided into the external and internal quality. The external quality of fruit mainly includes single fruit weight, fruit shape index, and color, which directly affect consumers’ purchase [3]; the inner quality of fruit mainly includes bioactive substances, trace elements, sugar, and acid content, which affect the quality of the appearance of fruit [4]. In addition, among stone fruits, the development cycle of sweet cherries (50–70 days) is shorter than that of apples, peaches, plums, and other fruits [5]. Sweet cherry is an ideal plant for studying the dynamic changes in stone fruit development.

Plant hormones are key regulators during fruit development and play an important role in fruit development. Unlike ethylene-dependent fruits such as apples and bananas, sweet cherries are non-climacteric fruits, the ripening of which is mainly related to ABA [6]. Ren et al. found that 9-cis-epoxycarotenoid dioxygenase (NCED) plays an important role in sweet cherry ripening and is a key restricted structural gene for ABA biosynthesis [7]. A mutual relationship exists between plant hormones, so other hormones cannot be ignored. GA3 is involved in fruit development, altering the expression of ABA biosynthesis genes and positively regulating some genes that are positively related to fruit ripening [8]. Saracoglu et al. found that the use of MeJA could affect the fruit quality and bioactive components of sweet cherries and delay fruit ripening [9]. Understanding the accumulation patterns of phytohormones will help to understand their roles in fruit development better.

A series of changes during fruit development results from the joint action of multiple metabolites and genes. Wang et al. found that 162 metabolites were different in the process of affecting the coloring of cucumbers, resulting in the different colors of cucumbers [10]. Analysis of carotenoids in apricots during four developmental periods showed that β-carotene and (E/Z)-phytoene are mainly responsible for the differences in carotenoids [11]. Romas et al. found a correlation between the stiffness values of strawberries at different stages of development and the mRNA abundance levels of *FaEXPS9*, *FaEXPA4*, *FaXTH1*, and *FaPG1* [12]. *INV*, *SUS*, *SPS*, *HK*, and *FK* coordinately promote sucrose biosynthesis during pumpkin fruit development and play an important role in the accumulation of soluble sugar in pumpkins [13]. In addition, various transcription factors are closely related to fruit growth and development [14]. The NAC family of transcription factors not only participates in lateral root development and flower formation but also regulates leaf and fruit ripening and senescence. *PacbHLH13* and *PacbHLH74* transactivate the *PacANS* promoter and participate in the regulation of anthocyanin synthesis in sweet cherry fruits [15]. Yeast two-hybrid and BiFC analysis showed that *SmWRKY44* could interact with *SmMYB1* to promote eggplant anthocyanin biosynthesis via transient expression [16]. In grapes, the overexpression of *VvWRKY22* reduces the contents of sucrose, glucose, and fructose and regulates the expression levels of sugar and ABA-related genes. *VvWRKY22* can also interact with important kinase proteins related to glucose metabolism and participate in glucose accumulation in glucose [17].

The metabolome can be used to effectively analyze the functions of genes involved in different metabolic processes. Combined metabolome and transcriptome analysis can explore the relationship between genes and metabolites. In Umer’s research on watermelons, metabolome and transcriptome analysis were used to establish a network of key genes that regulate organic acid and sugar metabolism and finally to identify seven key candidate genes [18]. Wang et al. used the transcriptome and metabolome to study fig coloration, fig anthocyanins, flavonols, and procyanidin metabolites, as well as global transcriptional changes in fig secondary metabolic pathways and transcription factors during fruit ripening and quality formation [19]. Through combined transcriptional and metabolic analysis of phenolic acid biosynthesis in *Cyclocarya paliurus* leaves at different developmental stages, eight differentially expressed genes (DEGs), six differentially expressed metabolites (DEMs), and 128 transcripts were found to be involved in the phenolic acid biosynthesis pathway. Finally, a regulatory network for phenolic acid biosynthesis was established [20].

The process of plant development depends on a precise and complex dynamic regulation system, which involves physiological changes such as changes in quality and color. Metabolome and transcriptome association analysis can be used to explain many physiological responses [21]. In the present study, the quality and endogenous components of sweet cherry fruit during the development process were measured and subjected to metabolome and transcriptome analysis to detect the DEMs and DEGs that affect fruit quality during sweet cherry development. Furthermore, the tissue metabolism regulatory network in the main growth period was constructed, and the regulatory switching genes of key metabolites were accurately identified. The results also revealed the changes in endogenous substances and quality-related metabolic pathways and candidate genes during the development of sweet cherries, laying a foundation for the breeding of high-quality sweet cherry varieties.

## 2. Results

### 2.1. Changes in the Dynamics of Substance Accumulation during Sweet Cherry Development

**Figure 1:**
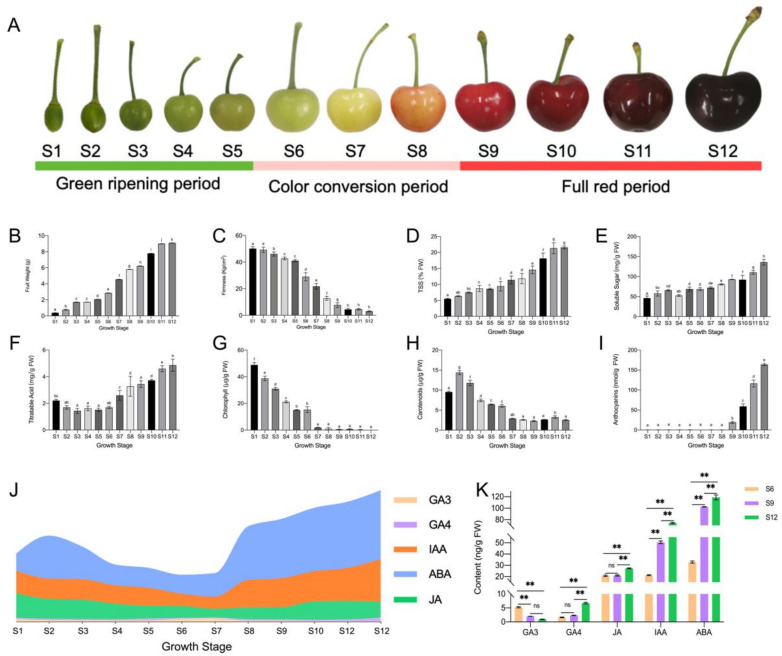
Changes in fruit quality and endogenous hormones during the development of sweet cherries. (**A**) Fruit growth and development status diagram; (**B**) single fruit weight, in which different lowercase letters indicate significant differences (*p* ≤ 0.05), the same below; (**C**) fruit firmness; (**D**) fruit soluble solids (TSS) content; (**E**) fruit soluble sugar content; (**F**) fruit titratable acid content; (**G**) fruit chlorophyll content; (**H**) fruit carotenoid content; (**I**) fruit anthocyanin content; (**J**) hormone profile during fruit development; (**K**) the specific changes in fruit endogenous hormones in the three periods of S6, S9, and S12, where ns means insignificant, and ** indicates an extremely significant correlation at the 0.01 level.

The changes in the developmental state and substance accumulation of sweet cherry fruit are shown in Figure 1. Throughout the developmental process, the fruit underwent transitions from small to large and from green to red (Figure 1A). As shown in Figure 1, the first dramatic change in single fruit weight, color, and other indicators occurred in the S6 period, and the second dramatic change occurred in the S9 period. Therefore, the 12 developmental periods were divided into three different stages, namely, the green ripening, color conversion, and full red period, according to the fruit developmental changes. Among these periods, the color conversion period was indicated as the starting point for drastic changes in fruit weight per fruit, soluble sugar, chlorophyll, anthocyanin, and hormones.

Sweet cherry single fruit weight increased continuously during the growth process and increased most rapidly during the color conversion period from 2.87 g to 9.00 g (Figure 1B). Fruit firmness continued to decrease as the fruit developed, with a more rapid decrease from the color conversion period (Figure 1C). Similarly, both fruit soluble solids (TSS, Figure 1D) and soluble sugars (Figure 1E) changed most significantly within the second and third stages. Changes in the titratable acid content of sweet cherry fruit initially decreased and then increased, reaching their maximum levels at the ripening stage (Figure 1F). The chlorophyll content was higher in the early stage and started to decline sharply to almost zero after entering the color conversion period (Figure 1G). By contrast, although the overall change in carotenoids was similar to that of chlorophyll, the decline was less dramatic than that of chlorophyll in the later stages, and some levels could still be detected (Figure 1H). Within the full red period, the anthocyanin content increased rapidly and finally reached 163.77 nmol/g FW, and the fruit gradually changed from white to deep purple-red (Figure 1I).

The hormone profile during sweet cherry fruit development is shown in Figure 1J. The ABA concentration remained high throughout the growth stage, and it first decreased and then increased. ABA was synthesized rapidly, mainly from the S7 period when the fruit started to change color (Figure 1A), and was the hormone with the largest proportion in sweet cherry fruit. The content of IAA showed a bimodal pattern, where it increased rapidly in S1 and S7 and increased gradually with fruit development, second only to ABA. The content of JA slowly decreased during fruit development and had the highest content in the S1 period, but the overall content change was small. GA3 and GA4 contents were both low in sweet cherry fruit development, but their trends were different. GA3 initially increased and then decreased, and it changed rapidly in the S5–S8 period. Therefore, GA3 mainly played a role in the color conversion period. By contrast, GA4 remained low in the early stage and increased rapidly to the highest value in the S11–S12 period.

The stages with large changes in hormone content were compared specifically (Figure 1K). The endogenous hormone content of sweet cherry fruit changed rapidly within these three growth stages. During the three periods, all hormone contents increased except for GA3, which reached its lowest level at the fruit ripening stage. The GA4 content showed a significant increase with fruit ripening to a large maximum. ABA content showed the most drastic change, from 32.91 ng/g FW to 119.05 ng/g FW. IAA content had the lowest level in the S6 period and increased by 3.48 times in the S12 period. The JA content changed very slowly, where its level remained at approximately 20 ng/g FW, suggesting that JA may not be acting at this stage.

### 2.2. Metabolome Analysis

The above results suggest that the color conversion period plays an important role in sweet cherry development. Therefore, we selected fruits from three periods (S6/start of color conversion, S9/end of color conversion, and S12/ripening state) for untargeted metabolomics (LC-MS/MS) analysis considering the changes in fruit indicators.

**Figure 2:**
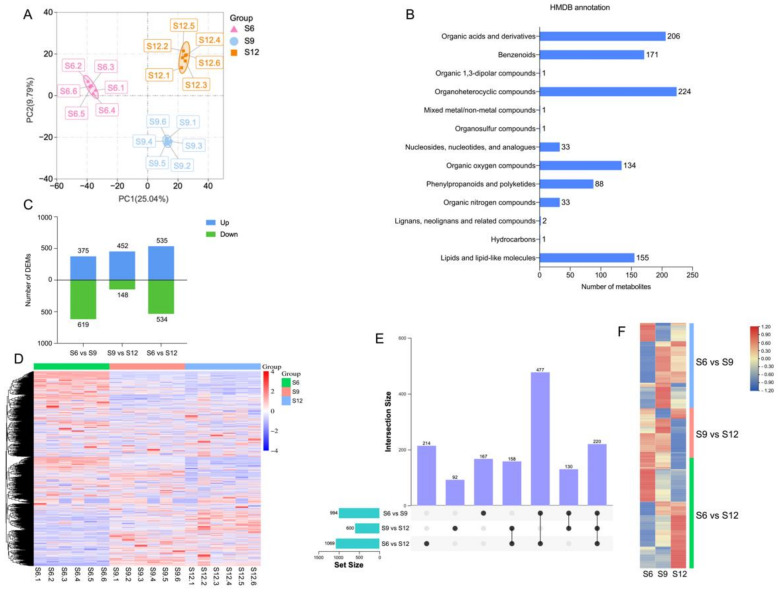
Metabolome analysis of sweet cherries during development. (**A**) PCA analysis of metabolites at each developmental stage; (**B**) HMDB classification annotation of metabolites; (**C**) the number of DEMs compared at any two different developmental stages; the number of upregulated and downregulated metabolites is represented by the bars above and below the *x*-axis, respectively; (**D**) heatmap of all DEMs at three developmental stages, with colors indicating the relative level content of each DEM, from low (purple) to high (red); (**E**) upset plots of ubiquitously and exclusively expressed DEMs in pairwise comparisons across developmental stages; (**F**) heatmap of expression patterns of unique DEMs in the three comparison groups, with different colors representing the relative level content of each DEM, from low (blue) to high (red).

The PCA results of metabolomic profiles are shown in Figure 2A, where the first two principal components were able to separate 18 samples, accounting for 25.04% and 9.79% of the total variability, respectively. In addition, the samples on the PCA analysis plot were divided into three groups, showing a trend of separation among metabolic groups and aggregation among replicates. These results indicate good reproducibility of the data during the experiment. Obvious differences were observed between the sweet cherry samples of the three periods. S9 and S12 were observed at the positive end of PC1, distributed at the positive and negative ends of PC2, respectively. The PLS-DA plots (Figure S1) also clearly distinguished the metabolic profiles of sweet cherry fruits in each period.

Metabolomic analysis identified 3079 metabolites, containing 1066 negative ions and 2013 positive ions (for details, see Table S6). The identified metabolites were annotated through HMDB classification (Figure 2B), and 1050 metabolites were classified into 13 groups. Among these metabolites, most metabolites belonged to the category of organoheterocyclic compounds (224 metabolites), accounting for 21.33% of all the annotated information, followed by organic acids and derivatives (206 metabolites), benzenoids (171 metabolites), lipids, lipid-like molecules (155 metabolites), organic oxygen compounds (134 metabolites), and phenylpropanoids and polyketides (88 metabolites).

Thresholds of VIP > 1.0, difference multiplier FC > 1.2 or FC < 0.833, and *p*-value < 0.05 were set to screen for DEMs. Comparisons were made between combinations of different periods, and the combination with the most DEMs was S6 compared with S12 (1069 in total, 535 upregulated, and 534 downregulated). The combination with the least differences was S9 vs. S12, with only 600 DEMs (452 upregulated and 148 downregulated, Figure 2C). A heat map of the 1458 DEM hierarchical clusters screened is shown in Figure 2D, indicating significant differences between combinations. Approximately 30% of the DEMs were highly expressed at S6, 20% were highly expressed at S9, 15% were highly expressed at S12, and 45% were similarly expressed in the three periods. A comparison of the two-two combinations of the three periods is shown in Figure 2E. A total of 220 DEMs were shared among the three combinations, and the most DEMs were shared between the two combinations of S6 vs. S9 and S6 vs. S12, with a total of 477 DEMs. A total of 167 DEMs were unique to S6 vs. S9, 92 DEMs were unique to S9 vs. S12, and 214 DEMs were unique to S6 vs. S12, indicating that the material changes were more intense in the S6 and S9 periods. The changes were more drastic. The expression of DEMs specific to the three combinations is shown in Figure 2F. The expression of these DEMs differed significantly among periods.

### 2.3. Transcriptome Identification

#### 2.3.1. Results of Overall Transcriptome Analysis

RNA-seq technology was used to study transcriptome data in different developmental periods of sweet cherries. A total of 441,209,814 raw data were generated from the three periods, and after filtering out splice sequences, uncertain reads, and low-quality reads, 402,434,278 high-quality clean reads were obtained, and an average of 92.62% of the clean reads were localized to the sweet cherry genome (see Table S2 for detailed results). A total of 31,642 transcripts were obtained, and the expression level of each gene was normalized to FPKM to obtain the full gene expression pattern during sweet cherry development (for details, see Table S7).

**Figure 3:**
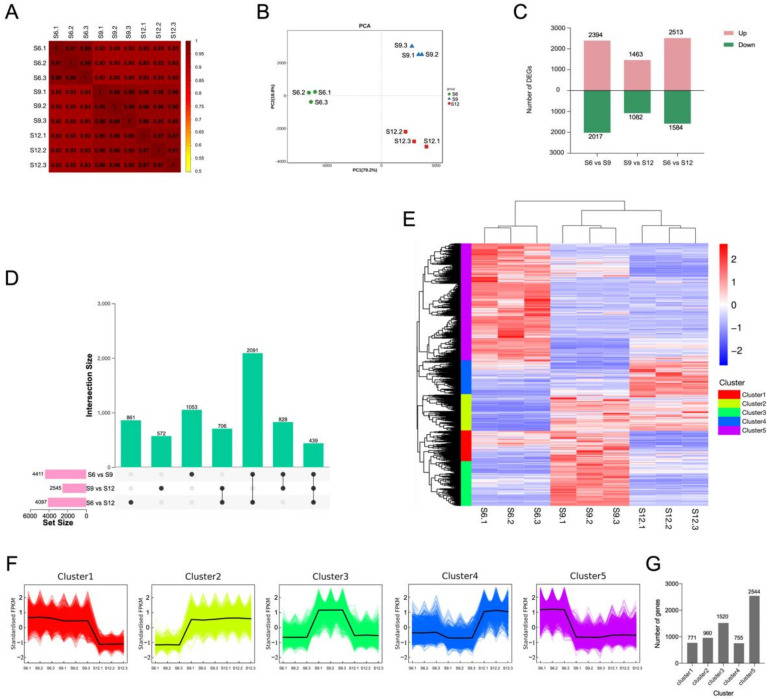
Transcriptome analysis of sweet cherries during development. (**A**) Spearman correlation coefficients of gene expression at different developmental stages; (**B**) PCA analysis of gene expression at each developmental stage; (**C**) the number of DEGs compared at any two different developmental stages; the number of upregulated and downregulated genes are represented by the bars above and below the *x*-axis, respectively; (**D**) upset plots of ubiquitously and exclusively expressed DEGs in pairwise comparisons across developmental stages; (**E**) hierarchical clustering of DEGs for all samples; (**F**) K-means clustering of DEGs expression trends, the expression profiles of genes in each cluster are represented in different colors, and the average expression profiles of all genes in each sample are represented in black; (**G**) summary of K-means clustering results, the specific number of DEGs contained in each cluster.

The correlation of gene expression levels among the samples is shown in Figure 3A. The high correlation between the three biological replicates at the same developmental stage indicates the differential expression of sweet cherry genes at different developmental periods. Notably, the gene expression between two samples (S9 and S12) was highly correlated, indicating that the differential expression of genes in the S6 period plays an important role in the later fruit development. PCA analysis was performed on the expression of the transcribed samples, and the results are shown in Figure 3B. The samples during the three developmental periods can be clearly distinguished on the score map, and the repeats were more closely focused, indicating differences in the gene expression of sweet cherries in different developmental periods. PC1 explained 79.2% of the features in the original dataset, and PC2 explained 18.8% of the features in the original dataset. PC1 showed a clear separation between the S6 period and the two other periods and a weak separation between S9 and S12. Therefore, similarly to the correlation results between samples, S9 and S12 had a more similar relationship than S6 to some extent.

#### 2.3.2. Identification of Differentially Expressed Genes

To investigate the transcriptional differences in samples from different developmental periods, we set a threshold *p*-value < 0.05 and |log2FoldChange| > 2 for differential gene screening, and 6550 DEGs were identified. The results of DEGs compared between different developmental periods are shown in Figure 3C. Among the three comparison groups, the S6 vs. S9 combination had the most DEGs at 4411. Overall, the number of upregulated DEGs was greater than the number of downregulated DEGs in all three comparison groups. The analysis of the common or unique DEGs among the three comparison groups is shown in Figure 3D. The lowest number (439) of genes was expressed in all three periods, and the highest number of genes (2091) was expressed in S6 vs. S9 and S6 vs. S12. Therefore, the transcriptome differential changes in fruits during S6 were the most significant among the three periods, and the transcriptional differences generated during S6 may be the main reason for the changes in fruit quality at a later stage; thus, they have a very important role in fruit development.

A hierarchical clustering heat map was generated for the three developmental periods, using FPKM values as expression levels (Figure 3E). The reproducibility within each period group was good, and the differences between the periods were significant, with S9 and S12 clustered together. The expression patterns of DEGs were divided into five groups, where S6 was mainly highly expressed in cluster 1 and part of cluster 4, S9, was mainly highly expressed in clusters 3, 4, and 5, and S12 was mainly highly expressed in clusters 2 and 3. The expression pattern of all DEGs in each sample was further analyzed by performing K-means clustering analysis (Figure 3F), and the specific number of DEGs in each grouping is shown in a bar graph. A total of 771 DEGs in cluster 1 were highly expressed during S6–S9. A total of 960 DEGs in cluster 2 were expressed mainly during S9–S12. Cluster 3 contained 1520 DEGs, accounting for 23% of the total DEGs, which were mainly expressed at S9, and lower expression was observed at S6 and S12. Cluster 3 and 5 contained most of the DEGs throughout the developmental process, except for cluster 5, which contained 2544 DEGs, accounting for 39% of the total DEGs, which were mainly expressed at S6. Clusters 3 and 5 contained the majority of DEGs throughout development. In addition, the expression profiles of most DEGs changed during the S6–S9 period, suggesting that the expression differences generated during the S6–S9 period mainly caused the subsequent quality changes.

#### 2.3.3. GO Enrichment Analysis

**Figure 4:**
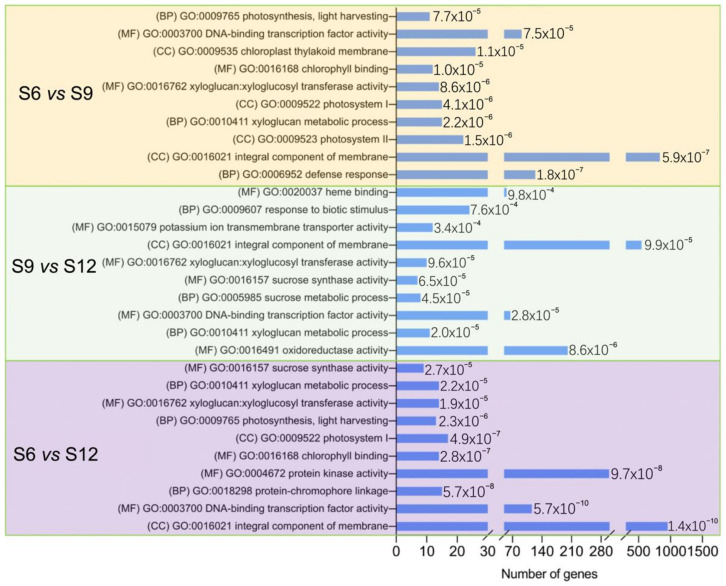
GO enrichment analysis of DEGs between groups was compared. The figure shows the top 10 rich items with extremely significant (≤0.05) *p*-values for each comparison, and the values of the *p*-values are shown in the bar chart. (BP): biological process; (MF): molecular function; (CC): cellular component.

The functions of DEGs were investigated using GO enrichment analysis, and the results are shown in Figure 4. The figure shows the names of the top 10 significant GO terms belonging to the three categories—biological process, molecular function, and cellular component—in the three comparison combinations. In all three comparison groups, most DEGs were significantly enriched in CC and MF, and the MF category was more enriched, indicating that these pathways play an important role in sweet cherry development. Notably, the S6 vs. S9 and S9 vs. S12 comparative groups were enriched for sucrose synthase activity, sucrose metabolic process, and other terms related to fruit sucrose. In both the S6 vs. S9 and S6 vs. S12 comparison groups, the terms photosynthesis, photosynthesis, light-harvesting, chlorophyll-binding, photosystem I, and chloroplast thylakoid membrane were enriched. This indicates that the fruit pigments started to change during S6, with a sharp decrease in chlorophyll (Figure 1G) and a rapid accumulation of anthocyanins (Figure 1I). Based on the results of GO enrichment analysis, the DEGs associated with quality changes during sweet cherry development could be further screened.

### 2.4. Transcription Factor Family Analysis

**Figure 5:**
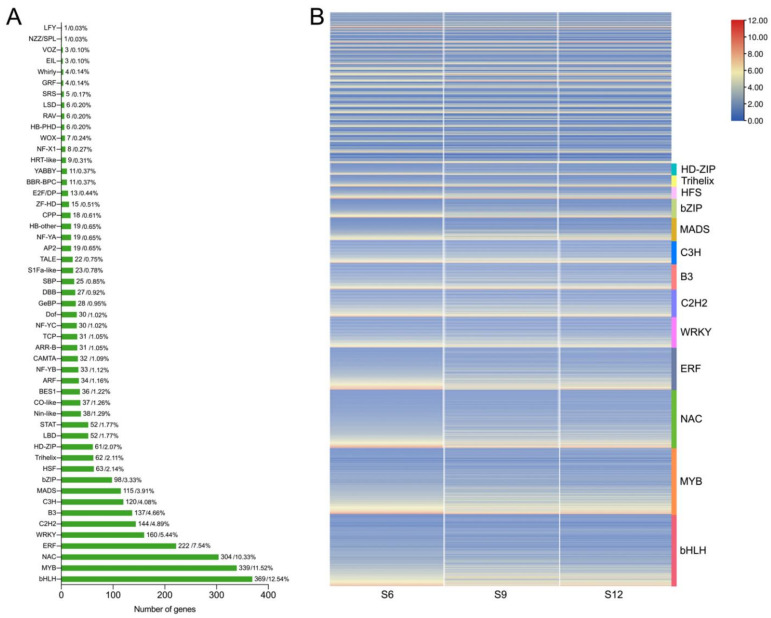
Analysis of transcription factors in different developmental stages of sweet cherries. (**A**) Statistical summary of transcription factor families; the number and proportion of each transcription factor family are displayed in a bar chart; (**B**) heat map of transcription factor expression patterns. The FPKM values of transcription factors were transformed according to log2.

In the transcription factor family analysis conducted, 2943 DEGs were identified as transcription factors, and these DEGs belonged to 51 transcription factor families. The transcription factor families mainly included bHLH, MYB, NAC, and WRKY (Figure 5A). Transcription factor families bHLH and MYB, which are related to the regulation of fruit coloration, occupied the highest percentages, at 12.54% and 11.52%, respectively. This was followed by a relatively high proportion of transcription factor families closely related to plant growth and development, such as NAC (10.33%), ERF (7.54%), WRKY (5.44%), B3 (4.66%), and bZIP (3.33%). Therefore, the expression patterns of these transcription factors were further analyzed, and the results are shown in Figure 5B. These transcription factors were expressed in all three periods, but the expression was higher in the S6 period than in the S9 and S12 periods, and these transcription factors may play an important regulatory role in fruit development.

### 2.5. Transcriptome and Metabolome Pathway Enrichment Analysis

**Figure 6:**
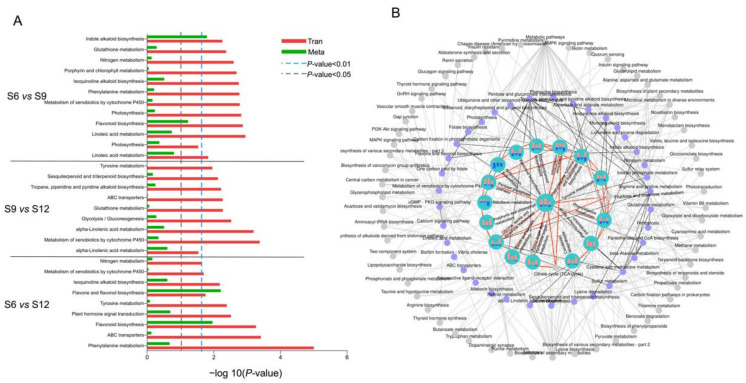
Annotation analysis of KEGG pathway in different developmental stages of sweet cherries. (**A**) KEGG-enriched top 10 pathway analysis of DEGs between each comparison group. Select differential genes with *p*-values ≤ 0.5 for KEGG enrichment analysis; (**B**) association analysis of each pathway. Different dots indicate different metabolic pathways: gray indicates pathways that were associated with pathways but not enriched in the top 30 or not enriched; purple indicates pathways that were enriched into the top 30 at least in one set of comparison groups; blue circles indicate pathways that were enriched in the top 30 and more closely linked, at least in one comparison group. The bars in the blue circles represent the number of DEMs and DEGs in three different developmental stages, respectively, red represents the number of DEGs contained in the pathway, and blue represents the number of DEMs in the pathway.

The metabolic pathways that were significantly enriched by DEGs, together with DEMs, were identified via KEGG pathway analysis. The top 10 significantly enriched metabolic pathways were mapped for each comparison group, in which 18 pathways were identified, and the results are shown in Figure 6A. In the comparison group of S6 vs. S9, pathways containing linoleic acid metabolism, flavonoid biosynthesis, indole alkaloid biosynthesis, and porphyrin and chlorophyll metabolism could be seen. Among these pathways, the enrichment of porphyrin and chlorophyll metabolism and flavonoid biosynthesis indicated that the chlorophyll in the fruit was gradually fading at this time, preparing for further conversion to anthocyanins. Moreover, the enrichment of the glycolysis/gluconeogenesis pathway was significant in the comparison group of S9 vs. S12, where glucose was gradually broken down, and a large number of precursors were produced to further promote the synthesis of anthocyanins, flavonoids, and alkaloids. Similarly, the flavone and flavonol biosynthesis, phenylalanine metabolism, and flavonoid biosynthesis pathways showed significant results in the S6 vs. S12 comparator group.

The association between the pathways was further analyzed by plotting the top 30 pathways that were significantly enriched in the three combinations in association diagrams, with complex and tight connections between the pathways, and the results are shown in Figure 6B. A total of 53 different pathways were enriched, among which pathways closely related to sweet cherry growth were remarkably enriched. As shown in the figure, the central role of the glycolysis/gluconeogenesis pathway was not only directly linked to several significantly enriched pathways, but also contained more DEGs and DEMs themselves. Glycolysis links various pathways of sweet cherry growth and development. First, it is directly involved in the formation and conversion of fruit sugars by linking amino sugar and nucleotide sugar metabolism, fructose and mannose metabolism, and starch and sucrose metabolism, which lay the foundation for the synthesis of other substances. In addition, it can directly connect the amino acid synthesis pathways such as cysteine and methionine metabolism and glycine, serine, and threonine metabolism, and then participate in the anabolic process of chlorophyll, endogenous hormones, and alkaloids in fruits. Moreover, glycolysis can also indirectly participate in fruit flavonoid and anthocyanin synthesis through phenylalanine, tyrosine, and tryptophan biosynthesis pathways. In addition, DEGs were abundantly enriched in flavonoid biosynthesis, phenylpropanoid biosynthesis, amino sugar, and nucleotide sugar metabolism pathways. The two comparison groups, namely, S6 vs. S9 and S6 vs. S12 had more DEGs and DEMs in most pathways than S9 vs. S12, further suggesting that metabolic transcriptional differences arising during the S6 period of development remarkably affect quality changes during fruit development.

#### 2.5.1. Analysis of the ABA Synthesis Pathway

**Figure 7:**
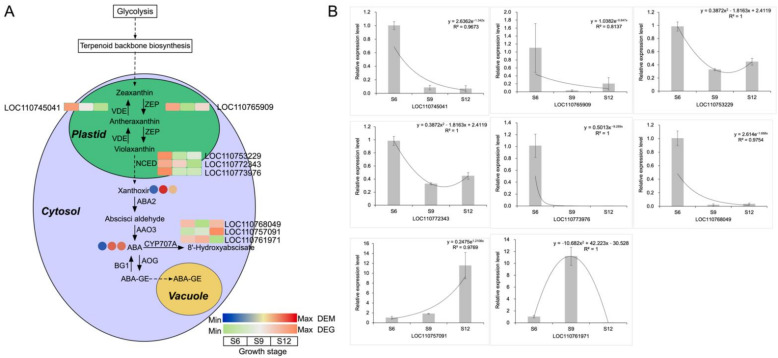
ABA synthesis pathway and gene expression analysis. (**A**) Schematic representation of the core pathway of ABA biosynthesis. The circle heat map represents the expression of DEMs in the three developmental stages, and the square heat map represents the expression of DEGs in the three developmental stages. (**B**) qRT-PCR analysis of ABA biosynthesis-related genes.

The hormonal analysis revealed that ABA showed the greatest variation during fruit development and accounted for the largest proportion, playing an important role in fruit ripening (Figure 1J). Moreover, the carotenoid biosynthesis pathway, which is the biosynthetic pathway of ABA, was remarkably enriched in Figure 6. Therefore, a detailed pathway analysis was carried out for this pathway, and the results are shown in Figure 7A and Table S3. With fruit development, the ABA content increased rapidly by 2.5-fold at S9 compared with S6. The high expression of zeaxanthin epoxidase (*ZEP*) and *NCED* genes in the synthetic pathway in the S6 stage promoted the formation of intermediate substances such as violaxanthin and flavin, which laid the material foundation for the massive accumulation of ABA in the later stage. The abscisic acid 8′-hydroxylase (*CYP707A*) gene downstream of the ABA synthesis pathway was highly expressed at S12, which catalyzed the formation of 8′-hydroxyabscisate from ABA. This finding indicates that the fruit had reached maturity at this time, and excessive ABA would be gradually degraded to produce other substances. To verify the validity of the transcripts, we performed qRT-PCR analysis on the DEGs of the all ABA synthesis pathways, and the results are shown in Figure 7B. The gene expression patterns were in general agreement with the transcriptome heat map, indicating that the results obtained from the transcriptome were relatively accurate and reliable.

#### 2.5.2. Metabolic Pathway Analysis

**Figure 8:**
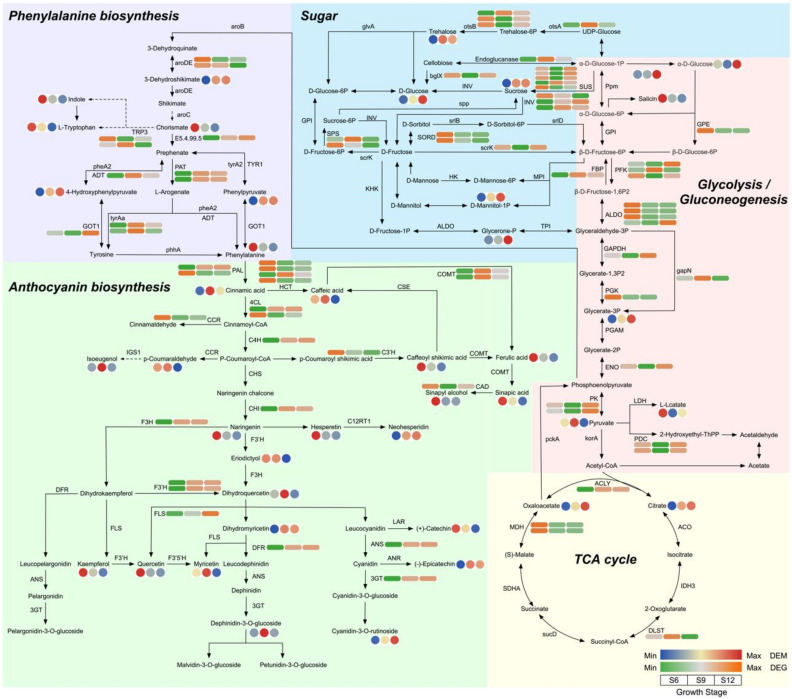
Metabolic pathway analysis centered on glycolysis/gluconeogenesis. The circle and square heatmaps represent the expression of DEMs and DEGs at three developmental stages, respectively.

To further analyze the significantly enriched pathways, we performed detailed pathway mapping, centered on the glycolysis/gluconeogenesis pathway, which mainly included the glycolysis, glycolysis/gluconeogenesis, phenylalanine metabolism, anthocyanin biosynthesis, and citrate cycle (TCA cycle) pathways, containing 42 DEMs and 73 DEGs (Figure 8 and Table S4).

During sugar and organic acid metabolism (including 13 DEMs and 40 DEGs), the expression of most DEMs increased with fruit development, including metabolites such as citric acid, trehalose, and alpha-D-glucose. Among these compounds, D-glucose metabolite expression was highest at S12, with a 1.89-fold increase compared with S6. Two DEMs were expressed at the highest level at S6 (salicin; (S)-lactate), and one DEM was expressed at the highest level at S9 (pyruvic acid). The contents of sugar components such as alpha-D-glucose, D-glucose, and sucrose were all at the highest level at S12. Among these compounds, D-glucose showed the most significant changes, indicating that it might be the most important sugar component that affects flavor changes in sweet cherries. The transcript levels of the structural genes sucrose synthase (*SUS*) and beta-fructofuranosidase (*INV*), which are closely related to D-glucose, mostly increased significantly from the S6 to S12 period. Two sucrose-phosphate synthases (*SPS*) and two L-iditol 2-dehydrogenase (*SORD*) were both expressed at the highest level at S6 and S9, promoting the conversion of fructose to D-Glucose. Similarly, three fructose-bisphosphate aldolases (*ALDO*), one putative glucose-6-phosphate 1-epimerase (*GEP*), one phosphoglycerate kinase (*PGK)*, and two malate dehydrogenase (*MDH*) all had the highest expression at the S6 period, and then remained at lower levels, showing the same trends as the metabolites.

The phenylalanine metabolism pathway involved seven DEMs and 11 DEGs. Four DEMs, namely, indole, L-tryptophan, chorismate, and phenylalanine, were highly expressed mainly in the S6 period. Particularly, phenylalanine expression decreased by 21.53-fold in the S12 period. All three DEMs (3-dehydroshikimate, phenylpyruvate, and 4-hydroxyphenylpyruvate) showed the highest expression in the S12 period. Most of the structural genes were highly expressed in the S9 and S12 periods.

Regarding the biosynthetic pathways of flavonoids and anthocyanins (21 DEMs and 22 DEGs), cyanidin-3-rutinoside, which is associated with fruit color, had the highest expression at S12, suggesting that it may be one of the main pigments that determines the color of sweet cherry fruit. Neohesperidin, dihydromyricetin, and epicatechin showed similar patterns of variation to cyanidin-3-rutinoside. In addition, 12 DEMs (phenylalanine, caffeoyl shikimic acid, (+)-catechin, kaempferol, hesperetin, naringenin, quercetin, eriodictyol, (E)-3-(4-hydroxyphenyl)-2-propenal, ferulic acid, sinapic acid, and sinapyl alcohol) were expressed at the highest level at S6 and six DEMs (cinnamic acid, caffeic acid, dihydroquercetin, myricetin, delphinidin 3-glucoside, and isoeugenol) at the highest level at S9. The expression of DEGs, namely, chalcone isomerase (*CHI*), flavonoid 3′-monooxygenase (*F3′H*), flavonol synthase (*FLS*), anthocyanidin synthase (*ANS*), dihydroflavonol 4-reductase (*DFR*), naringenin 3-dioxygenase (*F3H*), and anthocyanidin 3-O-glucosyltransferase (*3GT*), which are involved in the process from naringenin chalcone onwards in the pathway, gradually increased, consistently with the trends of the metabolites, thus further promoting pigment accumulation at a later stage and regulating sweet cherry anthocyanin accumulation. Overall, the intermediates of sugar synthesis were upregulated, the precursors of anthocyanin synthesis were upregulated during S6, and the anthocyanin synthesis pathway was upregulated during S9 and S12 in the development of sweet cherries.

### 2.6. Correlation Network Analysis and Validation

#### 2.6.1. Identification and Validation of Glucose Anabolic Correlation Networks

**Figure 9:**
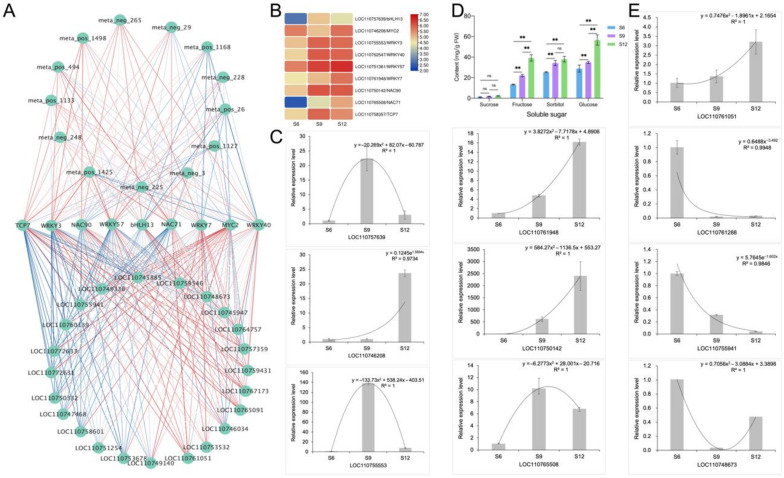
Identification and validation of glucose metabolism-related networks. (**A**) Correlation network identification of 9 transcription factors with DEMs and DEGs related to sugar anabolism. Correlations are represented by blue and red lines from low to high. (**B**) Expression patterns of 9 transcription factors, transformed according to log2 to FPKM values of transcription factors, with colors indicating the relative content of each transcription factor, from low (blue) to high (red). (**C**) qRT-PCR analysis of 6 transcription factors. (**D**) Composition and content of soluble sugars in different developmental stages of sweet cherries. ns means not significant, ** means extremely significant correlation at the 0.01 level; (**E**) qRT-PCR analysis of four sugar anabolic genes.

For further analysis and identification of transcription factors that regulate sugar accumulation in sweet cherries, screening within clusters 2 and 4 identified nine transcription factors, namely, *bHLH13*, *MYC2*, *WRKY3*, *WRKY40*, *WRKY57*, *WRKY7*, *NAC90*, *NAC71*, and *TCP7*, of which the expression patterns and specific information are shown in Figure 9B and Table S5. Therefore, the DEMs and DEGs related to sugar anabolism were subjected to correlation network analysis with these nine transcription factors, and the results are shown in Figure 9A. These transcription factors demonstrated a strong relationship with these DEMs and DEGs. *NAC71* was positively correlated with D-glucose and sucrose, and the correlations of *NAC71* with INV, *SUS*, and *SPS* were high, indicating that *NAC71* may be a potential positive regulator of fruit sugar metabolism accumulation. Moreover, *WRKY57* was positively correlated with most of the DEMs, and the correlations with SPS, *INV*, and *SUS* were also high, regulating fruit sugar metabolism. *WRKY7* and *MYC2* were also closely related to sugar metabolism genes, and by regulating related genes, they in turn regulated the sugar content of fruits. *WRKY3* showed negative correlations with *SUS*, *SPS*, and *INV*, which influenced sugar accumulation in sweet cherry fruits.

To further analyze and verify the compositional changes in the sugar components, we determined the soluble sugar components contained in sweet cherries (Figure 9D) using HPLC. Each soluble sugar component showed an increasing trend during sweet cherry fruit development. The most abundant soluble sugar component was glucose, followed by sorbitol and then sucrose, whereas fructose was the least abundant and did not change significantly during the three periods. Moreover, the trend of each component was consistent with the change in soluble sugar content (Figure 1E), and fructose and glucose increased rapidly by 1.78 and 1.62 times during the S9–S12 period. By contrast, sorbitol did not change significantly during the S9–S12 period, indicating that the dramatic increase in the soluble sugar content of sweet cherry fruits in the late period was not related to it. Combined with the analysis of the fruit sugar biosynthesis pathway (Figure 8), the results show that glucose is the main component that determines the sweetness of sweet cherries and influences the flavor of sweet cherries.

To verify the validity of the transcripts, we randomly selected six of the nine abovementioned transcription factors (*LOC110757639*, *LOC110746208*, *LOC110755553*, *LOC110761948*, *LOC110750142*, and *LOC110765508*) for qRT-PCR analysis, and the results are shown in Figure 9C. Four structural genes related to sugar metabolism (*LOC110761051*, *LOC110761288*, *LOC110744941*, and *LOC110748673*) were also randomly selected for qRT-PCR analysis, and the analysis results are shown in Figure 9E. Overall, the qRT-PCR results for most of the transcription factors and structural genes were consistent with the transcriptome data, indicating a high confidence level in the transcript data.

#### 2.6.2. Identification and Characterization of Anthocyanin Synthesis Correlation Network

**Figure 10:**
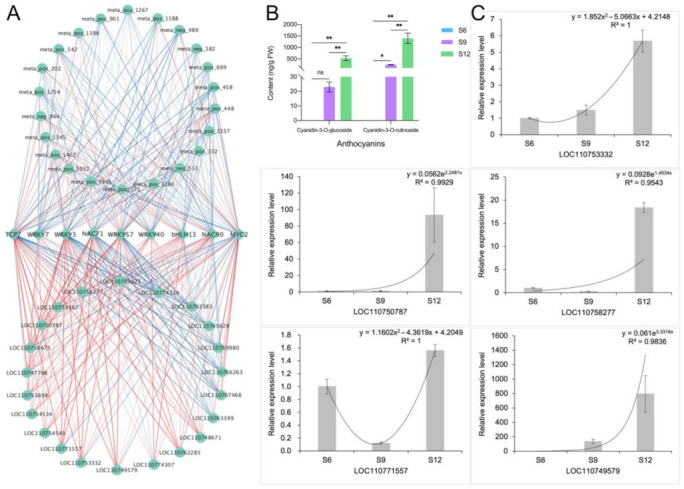
Identification and validation of anthocyanin synthesis correlation network. (**A**) Correlation network identification of 9 transcription factors and anthocyanin synthesis-related DEMs and DEGs. Correlations are represented by blue and red lines from low to high. (**B**) Composition and content of anthocyanins in different developmental stages of sweet cherries. ns means not significant, * indicates significant correlation at the 0.05 level, ** indicates extremely significant correlation at the 0.01 level; (**C**) qRT-PCR analysis of 5 anthocyanin synthesis-related genes.

The DEMs and DEGs related to anthocyanin synthesis were subjected to correlation network analysis with these nine transcription factors, and the results are shown in Figure 10A. Among them, *WRKY3*, *NAC71*, and *WRKY57* had the highest correlation with *DFR* and *3GT*, with correlation coefficients above 0.9, and they were also positively correlated with dihydroquercetin and cyanidin-3-O-rutinoside, which are important regulators of anthocyanin synthesis in sweet cherries. Both *bHLH13* and *WRKY7* were highly correlated with dihydromyricetin and positively correlated with *F3H, ANS*, and other genes which may play an important role in fruit coloration. *MYC2* had a high correlation with the anthocyanin pre-synthesis genes *PAL*, *4CL*, and *FLS*, indicating that it mainly plays a role in the flavonoid biosynthesis pathway. In conclusion, except for *MYC2*, most transcription factors were highly correlated with late anthocyanin synthesis genes.

The results of changes in the components of anthocyanins are consistent with the metabolome analysis (Figure 10B), in which the content of cyanidin-3-glucoside and cyanidin-3-rutinoside increased rapidly with development. Notably, the content of cyanidin-3-rutinoside was high and changed rapidly, and the content at maturity was approximately three times higher than that of cyanidin-3-glucoside. In conclusion, the results of the HPLC analyses were consistent with the findings of the metabolome analysis, indicating that cyanidin-3-rutinoside is the most important component that determines the dark purple color of sweet cherries at later stages.

Five structural genes related to anthocyanin synthesis, namely, *LOC110753332*, *LOC110749579*, *LOC110750787*, *LOC110771557*, and *LOC110758277*, were randomly selected for qRT-PCR analysis, and the results are shown in Figure 10C. The qRT-PCR results of these genes were consistent with the transcriptome data, indicating a high confidence level in the transcript data.

## 3. Discussion

**Figure 11:**
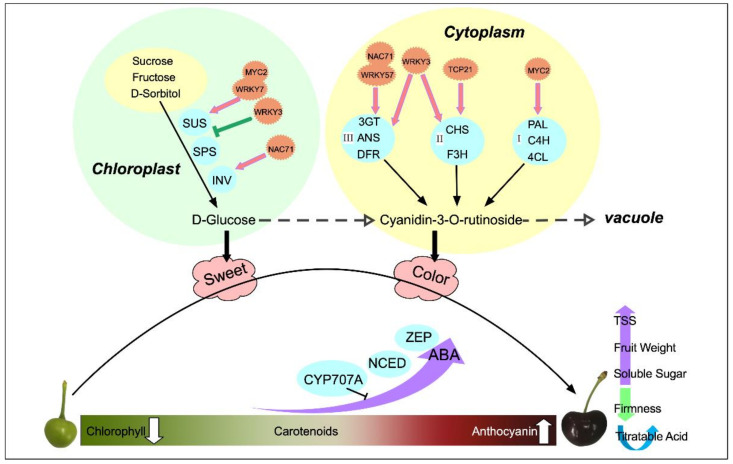
Schematic diagram of quality changes and regulation during the natural development of sweet cherries.

Fruit development and ripening is a very complex physiological and biochemical process, which involves a series of physiological and biochemical changes, including changes in fruit inclusions, synthesis, the decomposition of hormones, color, and other metabolic changes. During fruit development, the ripening process is important, and this stage determines the commercial value of the fruit. Moreover, the size, color, and flavor of the fruit in this stage are significant. Similarly to many drupes such as peaches and plums, sweet cherry fruit development follows a double ‘S’ growth pattern [22]. In the present study, the characteristics of single fruit weight and TSS, soluble sugars, and anthocyanin contents of sweet cherry fruit increased continuously with fruit development; their main change stages mainly occurred after color conversion periods; and their growth changes exhibited a double ‘S’ change pattern. Its specific change mode mechanism is shown in Figure 11.

Hormones are essential endogenous substances in the process of plant growth and development, which change with fruit development. Moreover, the changing pattern of hormones is consistent with the physiological changes of fruit. In sweet cherry fruits, the most abundant endogenous hormone was ABA, followed by IAA, which increased throughout the development, whereas JA and GA3, which have low abundance, showed an overall decreasing trend, which was consistent with previous studies [23]. The patterns of changes in GA3 and GA4 differed, suggesting the presence of different modes of action for the two compounds. GA3 was most active during the color conversion period, and GA3 promoted fruit expansion during this stage. Moreover, the exogenous application of GAs can improve the promotion of cell division and expansion to improve fruit size [24]. The content of GA4 tended to increase slowly at a later stage and its period of action may be different from that of GA3. By contrast, the study by Teribia et al. showed that GA4 content decreased with fruit development and was highly significantly correlated with anthocyanin, soluble solids, and fruit biomass [25]. The highest JA level was obtained at the beginning of fruit development. It showed an overall decreasing trend and it acted mainly in the pre-fruit stage. Similarly, during the development of apples, JA levels were higher in the early stages and then decreased, suggesting that JA is associated with the development of young fruits. In addition, Kondo et al. found that endogenous JA in sweet cherry promotes cell division and acts in the early stages [26]. The IAA generally decreased and then increased, reaching the peak in the late fruit stage. Similarly to tomatoes, the activity of IAA showed a bimodal pattern at the beginning of fruit development and the beginning of fruit ripening, indicating that IAA acts at fruit initiation [27]. Moreover, IAA can induce the synthesis of GAs at the initiation of fruit development and can co-regulate fruit development as an initiator of fruit development [28]. ABA is essential for the development of non-respiratory leapfrog fruit such as sweet cherries and strawberries [29]. In the present study, the ABA content was low in the early stage and increased dramatically from the S7 period, and this observation was mainly obtained in the color conversion period and full red period. In production, exogenous ABA is often applied to change the endogenous ABA content to regulate fruit development and promote endogenous anthocyanin biosynthesis, thus promoting fruit ripening [30]. However, ABA also promotes the ripening of respiratory leap fruits. Exogenous ABA treatment increases ABA content in tomato flesh and seeds, induces the expression of *ACS* and *ACO* genes, and promotes ethylene synthesis and fruit ripening [31]. The significant enrichment of carotenoid biosynthesis and significant changes in ABA content were observed in the metabolomic pathway studies conducted in this study, indicating the importance of ABA for sweet cherry development. In the ABA synthesis pathway, the higher expression of ZEP and NCED genes during S6 promoted the accumulation of xanthoxin, resulting in the synthesis of ABA. The high accumulation of ABA at the ripening stage promoted fruit ripening, and then the high expression of the *CYP707A* gene at the S12 period promoted ABA catabolism. Thus, *NCED, ZEP*, and *CYP707A* are the main genes that determine the ABA content of sweet cherries.

Sugar content is an important component that affects fruit flavor. It is a source of sweetness and energy for fruit tree growth and development; it also participates in regulating the fruit development process as a signaling substance [32]. The soluble sugar content of sweet cherries was consistent with the trend of TSS, which initially increased, then stabilized, and finally increased rapidly. The soluble sugars did not increase during the color conversion period, whereas anthocyanins started to accumulate, indicating that some sugars need to be consumed as precursors for anthocyanin synthesis and to provide energy [33]. The main soluble sugars in apricots, plumcots, and plums are glucose and fructose, whereas the sucrose content is very small and hardly accumulates in the early stage. In peaches, the sucrose content is the most important sugar component as the fruit grows [34]. D-glucose has a more central position in sweet cherries and can be converted by various pathways, using sucrose, fructose, sorbitol, and trehalose. D-glucose increased significantly in the S12 period, indicating that it was the most important soluble sugar component in sweet cherry fruit accumulation, and the results of HPLC confirmed this conclusion. In the tomato sugar biosynthesis pathway, *INV, SUS, SORD,* and *SPS* were significantly correlated with sugar content [35]. *SORD* promoted the conversion of sorbitol to fructose, which was catalyzed by *SPS* to sucrose, whereas α-D-glucose-1P was catalyzed by *SUS* to sucrose. Sucrose was then catabolized to glucose and fructose via *INV* [36]. In the present study, three *SUS* genes were highly expressed in the S12 period, and this condition promoted sucrose accumulation, whereas two *INV* genes were highly expressed at the S9 and S12 periods to promote the further conversion of sucrose to glucose and fructose accumulation. HPLC results showed that sorbitol content did not change significantly at the S9 and S12 periods, which was consistent with the downregulation of *SORD* expression.

Fruit color is an important indicator of fruit quality and maturity, with chlorophyll, carotenoids, and anthocyanins determining plant tissue color [37]. Sweet cherry fruits are in the green ripening stage from S1 to S5, and then the fruits rapidly change from green to white to yellowish. We observed a rapid decline in chlorophyll and carotenoids during this period, a stable concentration of carotenoids after S7, and a continuous decline in chlorophyll. This suggests that the yellowish color of the fruit during S7 may be caused by the continuous decrease in the chlorophyll content to a level lower than that of carotenoids and the green color gradually being replaced by yellow. At the onset of the color conversion period, the fruit started to accumulate a red color, the rapid accumulation of anthocyanins covered the yellow color caused by carotenoids, and the fruit gradually turned red until maturity. Anthocyanins determine the color of the fruit, and the types of anthocyanins contained in the fruit vary depending on the type of fruit. In strawberries, pelargonidin-3-glucoside is the main anthocyanin component [38]. In apples [39] and grapes [40], cyanidin 3-galactoside and malvidin-3-O-glucoside are the major anthocyanin components. In the present study, 21 DEMs were identified in the sweet cherry flavonoid and anthocyanin synthesis pathways, in which the most dramatic variation was observed in cyanidin-3-rutinoside. The content of cyanidin-3-rutinoside increased by 41.08-fold at S12 compared with S6 and later showed similar results based on the HPLC assay. The results of Blackhall et al. similarly indicated that cyanidin-3-rutinoside is one of the main components of sweet cherry anthocyanins [41]. The different stages in anthocyanin biosynthesis are regulated by various structural genes, receiving mainly *PAL*, *C4H,* and *4CL* in the early stages; *CHS, CHI*, and *F3H* in the middle stages; and *DFR, ANS,* and *3GT* in the later stages. In the present study, the phenylalanine content was gradually broken down with fruit development to produce cinnamic acid and other substances, accumulating sufficient precursor substances for anthocyanin synthesis. Among these compounds, *PAL*, *4CL*, and *CCR* were highly expressed in the S6 period, and their expression patterns were consistent with those of phenylalanine and *p*-coumaraldehyde. Structural genes such as *CHI, F3H*, *F3’H, DFR, ANS*, and *3GT* all showed high transcript levels at the maturation stage, and the qRT-PCR validation results were consistent with these findings, and the expression patterns were all consistent with anthocyanin accumulation. *CHI* and *CHS* are considered two decisive structural genes in the flavonoid biosynthesis pathway of *Phyllanthus emblica* (L.). These genes are closely related to fruit coloration, and the expression pattern increases with fruit development [42]. *PgCHS, PgF3H,* and *PgDFR* isolated in pomegranate are highly expressed in red fruits, and their transcript levels show a synchronous increase with the accumulation of flavonoids [43]. *C4H*, *4CL*, and *UFGT* play a decisive role in anthocyanin production in passionfruit [44].

Fruit development is regulated by structural genes, as well as multiple transcription factors. In the transcription factor analysis, 2943 DEGs were identified as transcription factors, and these DEGs belonged to 51 transcription factor families. A large number of transcription factor families, MYB, bHLH, WRKY, NAC, and bZIP, were related to fruit growth and development, and some of them were expressed at high and significant levels, suggesting their possible involvement in sweet cherry growth and development. Jin et al. found that *PavMYB10.1* of the MYB family may interact with *PavbHLH* and *PavWD40* and bind with the promoter regions of the anthocyanin biosynthesis genes *PavANS* and *PavUFGT* to participate in anthocyanin biosynthesis and determine the coloration of sweet cherries [45]. Transcription factors not only directly regulate the anabolism of structural genes to participate in fruit development, but also indirectly regulate fruit growth by participating in the regulation of phytohormone anabolism. *HpWRKY44* transcriptionally activates *HpCytP450-like1*, thus promoting dragon fruit coloration [46]. *FaRIF* of the NAC transcription factor family is a key regulator of strawberry fruit maturation from early developmental stages, controlling ABA biosynthesis and signaling, cell wall degradation and modification, and the phenol–propane pathway [47]. Wu et al. analyzed the bHLH transcription factor family of *Prunus mume* and concluded that bHLH genes are involved in organ and tissue formation in response to stressful environments, as well as in the biosynthesis of hormones and other secondary metabolites [48]. Similarly, the banana WRKY family is involved in ABA-induced cold tolerance by directly activating *NCED* expression to increase ABA levels [49]. The ERF family is involved in regulating tomato ripening by acting on ethylene biosynthesis [50], and bZIP may be involved in ripening senescence in pears by participating in the regulation of the biosynthetic pathways of ABA and MeJA [51]. These transcription factors affect plant growth and development overall by regulating hormone synthesis, sugar synthesis, and other pathways.

In the present study, nine transcription factors were initially identified. They belong to four transcription factor families, namely, bHLH, NAC, WRKY, and TCP. Correlation network identification results show that *NAC71* and *WRKY57* may promote the accumulation of D-glucose by activating the expression of *INV*, *SUS*, and *SPS* genes. *WRKY3* may be a negative regulator of sugar accumulation and may inhibit the expression of SUS. *MYC2* is mainly correlated with the pre-colored fruit genes *PAL*, *C4H*, and *4CL*, which accumulate a large number of precursors for anthocyanin synthesis. *TCP7* was more highly correlated with *CHI* and *F3H.* Furthermore, *NAC71*, *WRKY3*, and *WRKY57* mainly regulated late key structural genes such as *DFR*, *ANS*, and *3GT* to promote fruit coloration. The molecular mechanisms of specific transcription factors should be studied for the regulation of sweet cherry growth and development. However, the roles of multiple transcription factors in sweet cherry growth and development have not been completely studied. In our next studies, we will carry out an in-depth analysis of these transcription factors to identify their functions.

## 4. Materials and Methods

### 4.1. Plant Materials

In the present study, the ‘Hongdeng’ sweet cherry was used as the test material, collected from the sweet cherry test base in Hanyuan County, Ya’an City, Sichuan Province, China. Three sweet cherry fruit trees with good growing conditions and the same developmental period were selected, and sampling was carried out on 7 April 2021 (5 days after flowering). Sampling was carried out every three days until the fruit matured. Sampling was conducted from 9:00 am to 10:00 am, and samples were collected in 12 periods (S1, S2, S3, S4, S5, S6, S7, S8, S9, S10, S11, and S12), which were divided into three stages according to the fruit development and color change (green ripening period, color conversion period, and full red period) (Figure 1A). After harvesting, the fruit was immediately brought back to the laboratory for imaging and the measurement of single-fruit weight and firmness. Then, the samples were quickly cut into uniform slices, quickly frozen in liquid nitrogen, and stored in a −80 °C refrigerator for use in subsequent experiments. Three biological replicates were prepared for the samples in each period.

### 4.2. Physiological Parameters

Single-fruit weight was determined using an electronic balance. A GY-1 firmness tester was used to determine fruit firmness. Soluble solid content was determined using a MASTER-M hand-held saccharimeter. Soluble sugar content was determined using the anthrone colorimetric method. Titratable acid content was determined via acid-base titration. Chlorophylls and carotenoids were extracted using an acetone-alcohol mixture (*v*/*v* = 1:1), and absorbance was determined using a spectrophotometer. The anthocyanin content was determined using the 1% hydrochloric acid-methanol method. The endogenous hormone content of the fruits was determined using ELISA kits (Enzyme immunoassay, Yancheng, China). Three replicates were determined for each sample of data.

Both the soluble sugar component and anthocyanin component were determined by means of high-performance liquid chromatography (HPLC), using an Agilent 1260 II high-performance liquid chromatography system (Agilent Technologies Co. Ltd., Santa Clara, USA). The specific determination methods were as follows.

The soluble sugar component was determined as follows. The column was an Agilent (4.6 mm × 250 mm), 0.5 μm NH2 column with a refractive index detector. The mobile phase was acetonitrile:water (*v*/*v* = 80:20) at a flow rate of 1 mL·min^−1^. The column temperature was 25 °C, the detector temperature was 40 °C, and the injection volume was 10 μL.

The anthocyanin component was determined as follow. The column was a Comatex PR15 (Hamilton, Reno, NV, USA, 250 mm × 4.6 mm), 5 μm C18 column with a diode array detector. Mobile phase A was water, mobile phase B was acetonitrile, and mobile phase C was formic acid. The specific gradient elution procedures were: 0–4 min, 92% A, 6.4% B, 1.6% C; 4–13 min, 82.5% A, 15.9% B, 1.6% C; 13–20 min, 67.5% A, 30.9% B, 1.6% C. The flow rate was 1 mL·min^−1^, the injection volume was 10 μL, the column temperature was 30 °C, and the detection wavelength was 520 nm.

### 4.3. Metabolome Analysis

#### 4.3.1. Metabolome Extraction

The sample was accurately weighed at 100 mg, added to 0.6 mL of 2-chlorophenylalanine (4 ppm) methanol (−20 °C), and vortex-shaken for 30 s. Then, 100 mg of glass beads were added, placed in a tissue grinder for 60 s at 55 Hz, and sonicated at room temperature for 15 min. The supernatant was filtered through a 0.22 μm membrane, and the filtrate was added to the assay bottle. Approximately 20 μL of each sample to be tested was mixed into QC samples to correct for deviations in the analysis results of the mixed samples and errors caused by the analytical instrument. The remaining samples were used for ultra-performance liquid chromatography tandem mass spectrometry (UPLC-MS/MS) analysis.

#### 4.3.2. Metabolite Detection and Analysis

The chromatographic conditions were as follows. We used an ACQUITY UPLC^®^ HSS T3 1.8 μm (2.1 × 150 mm) column with an autosampler set at 8 °C, and 2 μL of the sample was injected at a flow rate of 0.25 mL/min and a column temperature of 40 °C. The mobile phases were positive and negative ions, 0.1% formic acid in water (D), and 0.1% formic acid in acetonitrile (E). The gradient elution program was as follows: 0–1 min, 98% D, 2% E; 1–9 min, 74% D, 26% E; 9–12 min, 26% D, 74% E; 12–13.5 min, 2% D, 98% E; 13.5–14 min, 50% D, 50% E; 14–20 min, 98% D, 2% E.

The mass spectrometry conditions were as follows. We used an electrospray ionization source (ESI) (Agilent Technologies Co. Ltd., Santa Clara, USA) in simultaneous positive and negative ionization acquisition modes. The spray voltage was ±3.50 kV, the sheath gas flow rate was 30 arb, the auxiliary gas flow rate was 10 arb, and the capillary temperature was 325 °C. A full scan was performed at a resolution of 60,000 with a scan range of 81–1000 m/z, and higher energy collision-induced dissociation was used for secondary cleavage with a collision voltage of 30 eV, and dynamic exclusion was used to remove unnecessary MS/MS information.

Raw data were pre-processed for the data search using Compound Discoverer 3.1 software. The data were first screened in terms of the retention time and mass-to-charge ratio, and peak alignment was performed. Then, the exact molecular weight of the compound was determined based on the mass-to-charge ratio in the high-resolution XIC plot, and molecular formula prediction was performed based on mass number deviation and adduct ion information. Using the high-resolution mass spectrometry (HRMS) detection technique, molecular characteristics in the samples were detected as much as possible. The molecular characteristic peaks were identified by matching the high-quality mzCloud database (https://www.mzcloud.org/ (accessed on 1 November 2021)) and the mzVault and MassList databases constructed from the standards. The compounds with the coefficient of variance values less than 30% in the QC samples were then selected as the final identification results for subsequent analysis.

Next, multivariate statistical analyses of metabolites, including principal component analysis (PCA) and partial least squares discrimination analysis (PLS-DA) were performed to reveal differences in metabolites across groups. The data were log-transformed and centrally formatted using MetaX software (http://metax.genomics.cn/ (accessed on 15 November 2021)). Hierarchical clustering (HCA) and metabolite correlation analysis were performed to reveal the relationships between samples and between metabolites and metabolites. The identified metabolites were functionally and taxonomically annotated with major databases, including the Kyoto Encyclopedia of Genes and Genomes (KEGG, http://www.genome.jp/kegg/ (accessed on 5 December 2021)) and the Human Metabolome Database (HMDB, https://hmdb.ca/ (accessed on 6 December 2021)), to understand the functional properties and classifications of different metabolites.

The variable importance in the projection (VIP) values and fold change (FC) of the first principal component of the PLS-DA model was used and combined with *p*-values from the *t*-test to determine differentially expressed metabolites. The threshold values were set as VIP > 1.0, FC > 1.2 or FC < 0.833, and *p*-values < 0.05.

Finally, the biological significance of metabolite correlation was explained via functional analysis, such as metabolic pathway analysis. At a threshold of *p*-values ≤ 0.05, a KEGG term that met this condition was defined as a KEGG term that was significantly enriched in DEMs. The main biological functions exercised by DEMs can be determined by means of KEGG significance analysis.

### 4.4. Transcriptome Sequencing

#### 4.4.1. RNA Extraction and Transcriptome Sequencing Library Preparation

Total RNA was extracted using a total RNA kit (TIANGEN Biotech, Beijing, China). The concentration and purity of RNA samples were checked using a NanoDrop 2000 spectrophotometer (Thermo Fisher Scientific, Waltham, MA, USA), and the integrity of RNA samples was checked using an Agilent 2100 Bioanalyzer and 2100 RNA Nano 6000 assay kit (Agilent Technologies, Co. Ltd., Santa Clara, CA, USA). Oligo(dT) magnetic beads were used to enrich mRNA with polyA tails. mRNA was then randomly interrupted by divalent cations in the NEB fragmentation buffer. The fragmented mRNA template and random oligonucleotide primers were used as raw materials to synthesize the first strand of cDNA. Next, the second strand of cDNA was synthesized using the RNaseH component and DNA polymerase I with dNTPs as the raw material, and the double-stranded cDNA fragments were purified and recovered. After purification of the double-stranded cDNA, end repair and A-tailing were used to connect the sequencing junction, and the cDNA with a length of approximately 200 bp was amplified by PCR with AMPure XP beads, the PCR products were purified with AMPure XP beads, and the libraries were constructed. Initial quantification was carried out using a Qubit 2.0 Fluorometer, and the library was diluted to 1.5 ng/µL. The constructed library was quality-checked using an Agilent 2100 bioanalyzer (Agilent Technologies, Co. Ltd., Santa Clara, CA, USA), and when the insert size reached the expected level, qRT-PCR was performed to accurately quantify the effective concentration of the library to ensure the quality of the library. After the libraries passed the check, Illumina sequencing was performed on each library to generate 150 bp paired-end reads according to the effective concentration and the need for target next-level data.

#### 4.4.2. Data Processing and Analysis

The raw data were filtered using Trimmomatic software to remove the reads with connectors (adapter). Reads with a proportion of N (N indicates that the base information cannot be determined) greater than 10% and low-quality reads (reads with the number of bases with Qphred ≤ 20 accounting for more than 50% of the whole read) were removed to obtain clean data. The pre-processed data were compared to the reference genome using HISAT2 software to obtain the localization information of the sequencing data on the reference genome. The reference genome and gene model annotation files were downloaded directly from the NCBI website (https://www.ncbi.nlm.nih.gov/ (accessed on 15 November 2021)). New transcripts were predicted using StringTie software.

The transcript abundance of all single genes in each sample was calculated using Htseq-count (v0.6.0). The expected number of fragments per kilobase of transcript sequence per millions (FPKM) of base pairs of sequenced values was used to analyze the expression levels of genes. The samples were analyzed for differential expression using DESeq2 v1.22.1, the readcount data were normalized, and the *p*-values were corrected using Benjamini and Hochberg methods. *p*-values and |log2foldchange| were used as criteria to determine significant differential expression. Thresholds of *p*-values < 0.05 and |log2FoldChange| > 2 were set for differential gene screening.

Gene Ontology (GO) enrichment analysis was performed using the topGO R package. GO analysis corrects for gene length bias, clarifies the biological function of the differential genes, and involves histogram plotting based on the results of analysis using GraphPad9 software. Kyoto Encyclopedia of Genes and Genomes (KEGG) analysis was performed using the clusterProfiler R package to clarify the signaling pathways involved in the differential genes.

The PlantTFDB database (http://planttfdb.gao-lab.org/ (accessed on 20 November 2021)) was used to screen and categorize the predicted possible transcription factors. The log2(FPKM) values of differentially expressed genes were then used for clustering heat map plotting using the TBtools software (http://www.tbtools.org/ (accessed on 15 January 2022)).

#### 4.4.3. Quantitative Real-Time PCR (qRT-PCR) Analysis

The qRT-PCR analysis was performed using a Bio-Rad CFX96TM Real-Time PCR System (Bio-Rad, Hercules, CA, USA) and 2 × TSINGKE^®^ Master qRT-PCR Mix (SYBR Green I) (TSINGKE, Beijing, China). The amplification procedure included pre-denaturation at 95 °C for 30 s, denaturation at 95 °C for 0.05 s, and annealing at 59 °C for 30 s. The number of amplification cycles was 39, and each sample was repeated three times. The internal reference gene was *ACTIN*. Gene-specific primers were designed using Primer Premier 6, and the primer sequences are shown in Table S1. Each 20 μL of the PCR reaction solution contained 2.0 µL of diluted cDNA, 0.8 µL of each primer (10 mM), 10 µL of SYBR Green I mix, and 6.4 µL of dd H_2_O. Finally, gene expression was calculated using the 2^−ΔΔCt^ method.

### 4.5. Combined Transcriptome and Metabolome Analysis

A joint analysis of metabolomic and transcriptomic data was performed. All DEGs and DEMs obtained were simultaneously mapped to the KEGG pathway database to obtain their common pathway enrichment information. Subsequently, the major biochemical pathways and signal transduction pathways in which DEM and DEG were jointly involved were identified. Histograms of the top 10 significantly enriched metabolic pathways in each comparison group were plotted, whereas the correlation of the top 30 significantly enriched pathways in the three combinations were mapped using the OmicShare tool (https://www.omicshare.com/tools/ (accessed on 20 January 2022)). The DEMs obtained from the analysis were correlated with DEGs based on Pearson correlation coefficients to measure the degree of association between genes and metabolites, and correlation network plots were generated using Cytoscape v3.9.1 software.

## Data Availability

Transcriptional and metabolic data were generated by TSINGKE Biotechnology Co., Ltd., and physiological data were measured by the authors themselves. Transcriptome data are available at the National Genomics Data Center database under the accession number CRA007287 (https://ngdc.cncb.ac.cn/search/?dbId=gsa&q=CRA007287/ (accessed on 10 June 2022)).

## Supplementary Materials

The following supporting information can be downloaded at: https://www.mdpi.com/article/10.3390/ijms23137402/s1.
