# Supplementary figures and images for: Transcriptomic and Metabolomic Analysis of Quality Changes during Sweet Cherry Fruit Development and Mining of Related Genes
Source: Int J Mol Sci. 2022 Jul 3;23(13):7402. doi: 10.3390/ijms23137402 (PMC9266358; doi:10.3390/ijms23137402)

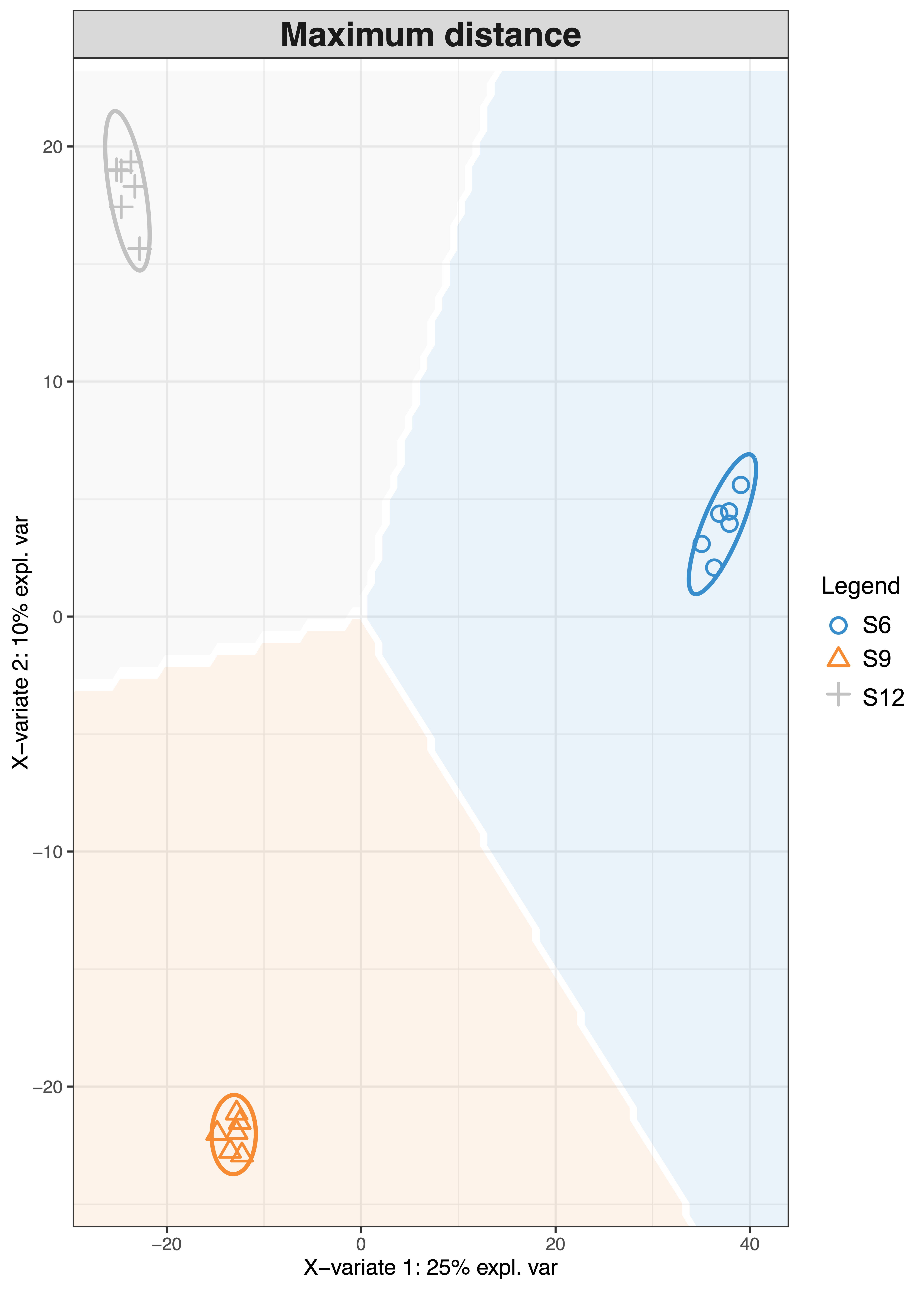

Supplement: Supplementary file 1 [file ijms-23-07402-s001.zip › Figure S1.tiff]
